# Hypermature Cataract With Secondary Glaucoma: A Case Report

**DOI:** 10.7759/cureus.106626

**Published:** 2026-04-08

**Authors:** Muralidhar Parri, Mansi D Devalla

**Affiliations:** 1 Ophthalmology, All India Institute of Medical Sciences, Mangalagiri, Mangalagiri, IND

**Keywords:** cortex aspiration, intraocular lens, intraocular pressure, milky white appearance, phacolytic glaucoma

## Abstract

Hypermature cataracts are uncommon in modern ophthalmic practice due to earlier surgical intervention. When complicated by phacolytic glaucoma, they present as an ophthalmic emergency requiring prompt management. An elderly female presented with pain and vision loss in the right eye for one week. Examination revealed hand-movement vision, intraocular pressure (IOP) of 54 mmHg, and a milky white cornea obscuring anterior segment details. Ultrasound B-scan showed a normal posterior segment. IOP was reduced with intravenous mannitol and topical therapy. Cataract extraction was performed via a sclerocorneal tunnel with trypan blue staining, nucleus prolapse, and aspiration. Intraoperative findings included a broken anterior capsule and zonular dialysis. A three-piece intraocular lens was implanted in the sulcus. Postoperatively, IOP normalized, corneal clarity improved, and vision recovered to 6/12 by the fourth week. This report highlights the importance of timely recognition and management of a hypermature cataract with secondary glaucoma. Despite advanced pathology and intraoperative challenges, tailored surgical techniques and appropriate lens placement can achieve favorable outcomes.

## Introduction

Cataract remains the leading cause of reversible blindness worldwide and continues to be a major public health concern, particularly in developing countries [1]. Although advances in surgical techniques and increased accessibility of cataract surgery have reduced the incidence of advanced cataracts, delayed presentation still occurs in certain populations. As a result, hypermature cataracts and their associated complications may still be encountered in clinical practice. A hypermature cataract represents the final stage of lens degeneration. At this stage, liquefaction of the cortical material occurs, and the lens capsule becomes fragile and permeable. Degenerative changes within the lens may lead to leakage of high-molecular-weight lens proteins into the anterior chamber. This process can result in lens-induced glaucoma, particularly phacolytic glaucoma [2]. Phacolytic glaucoma is characterized by obstruction of the trabecular meshwork by lens proteins and macrophages that ingest these proteins. The resulting impairment of aqueous humor outflow leads to markedly elevated intraocular pressure (IOP). Patients often present with acute ocular pain, redness, and severe visual loss. Corneal edema caused by elevated IOP may obscure anterior segment structures, making clinical evaluation difficult.

The management of phacolytic glaucoma involves two essential components. The first step is rapid reduction of intraocular pressure to relieve symptoms and prevent optic nerve damage. Medical therapy typically includes hyperosmotic agents and topical aqueous suppressants. Once IOP has been controlled and corneal clarity improves sufficiently, definitive management involves removal of the cataractous lens [3]. Surgical management of hypermature cataracts can be technically challenging. The lens capsule may be fragile, cortical material may be liquefied, and zonular support may be compromised. Visualization may also be limited due to corneal edema or small pupil size. In such situations, intraoperative modifications, including capsule staining and alternative intraocular lens placement, may be required to ensure successful outcomes [4]. This case report describes the clinical presentation and management of an elderly female with a hypermature cataract complicated by secondary glaucoma. The report highlights the importance of early recognition of lens-induced glaucoma and demonstrates how appropriate surgical planning can result in favorable visual recovery even in complex cases.

## Case presentation

An elderly female patient presented with complaints of pain and loss of vision in the right eye for one week. The patient reported that vision in the affected eye had been gradually deteriorating over several years but had suddenly worsened over the past week, associated with significant ocular pain and redness. There was no history of trauma, previous ocular surgery, or similar symptoms in the fellow eye. On examination, visual acuity in the right eye was reduced to hand movements close to the face. The left eye had relatively better vision and did not show any acute signs.

**Figure 1:**
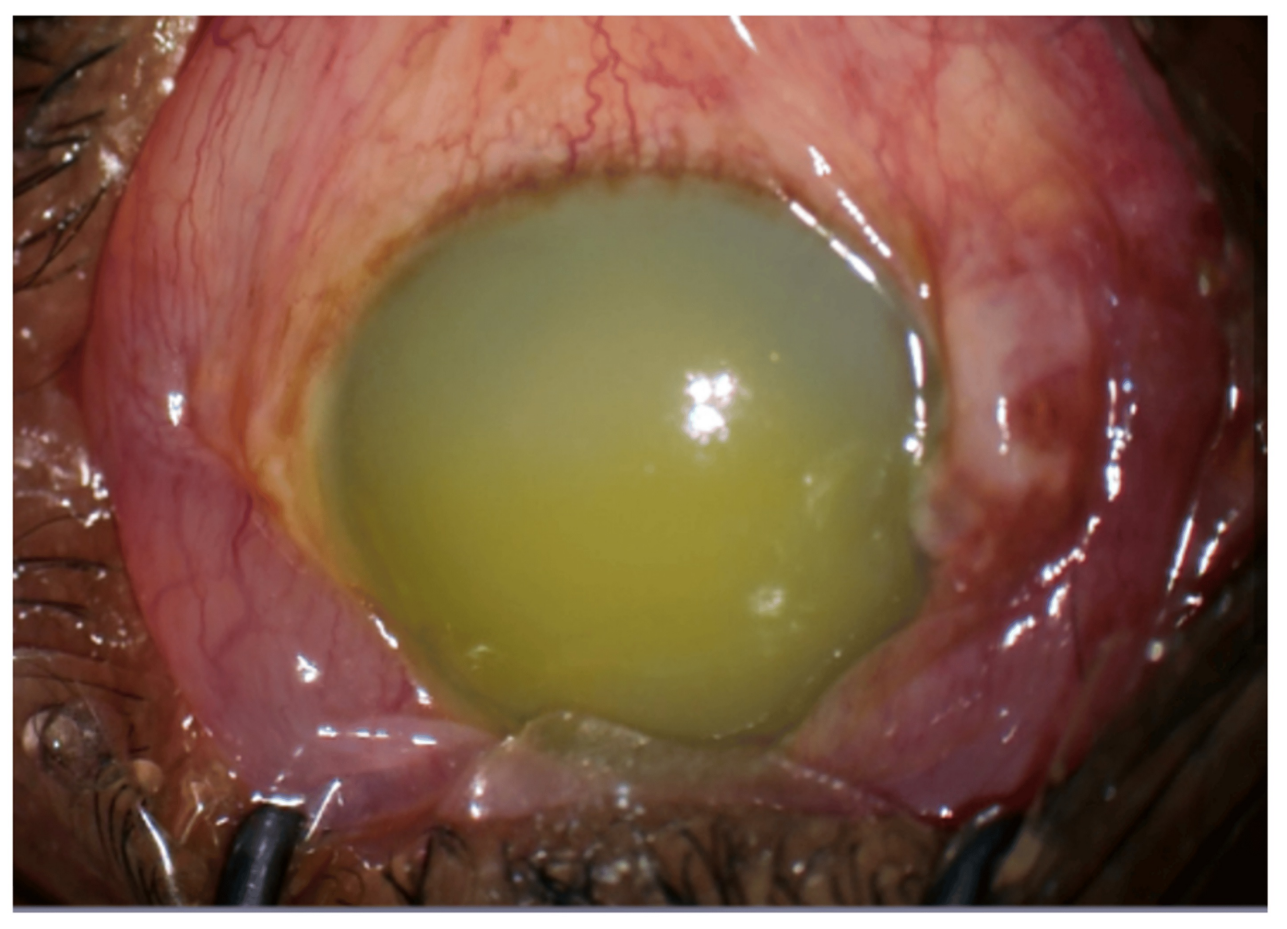
Intraoperative appearance of right eye showing liquified lens material in the anterior chamber

Slit-lamp examination revealed marked corneal edema and anterior chamber reaction with lens proteins in the right eye, giving a milky white appearance that obscured visualization of the anterior segment. Phacodonesis was noted, with an inferiorly sunken brownish nucleus in the capsular bag, suggestive of zonular weakness. Conjunctival congestion was present, and the eye appeared inflamed. Intraocular pressure in the right eye measured 54 mmHg, indicating severe ocular hypertension consistent with secondary glaucoma. Because of the opaque cornea, a B-scan ultrasonography was performed, which demonstrated a normal posterior segment with an attached retina and a clear vitreous cavity. Based on these findings, a diagnosis of hypermature cataract with secondary glaucoma (phacolytic glaucoma) was made. The intraoperative appearance of the right eye with liquefied lens material in the anterior chamber is shown in Figure 1.

The primary goal of initial management was to rapidly reduce IOP to relieve symptoms and prevent optic nerve damage. After systemic evaluation confirmed that osmotic therapy could be safely administered, the patient received intravenous 20% mannitol at a dose of 1 gm/kg. Topical medications were also initiated to suppress aqueous humor production and control inflammation: 1% Prednisolone acetate eye drops four times a day, and a combination of timolol and brimonidine eye drops twice daily. Following treatment, IOP decreased significantly from 54 mmHg to 21 mmHg, resulting in partial improvement of corneal clarity, which allowed better visualization of the anterior segment and facilitated definitive surgical planning.

Manual small incision cataract surgery (MSICS) was performed under peribulbar anesthesia using lignocaine. MSICS was the preferred choice over phacoemulsification in this case scenario because of the presence of a hypermature Morgagnian cataract, a hard nucleus, and zonular weakness. A 4 mm sclerocorneal tunnel incision was created to access the anterior chamber. A side port was made at the 9 o’clock position using a 15-degree blade. A saline wash of the anterior chamber contents was performed to remove inflammatory debris and lens proteins. Intraoperative findings included a small pupil, a broken anterior capsule, a hypermature cataract, and zonular dialysis involving approximately two clock hours. Trypan blue staining was used to enhance the visualization of the capsule.

**Figure 2:**
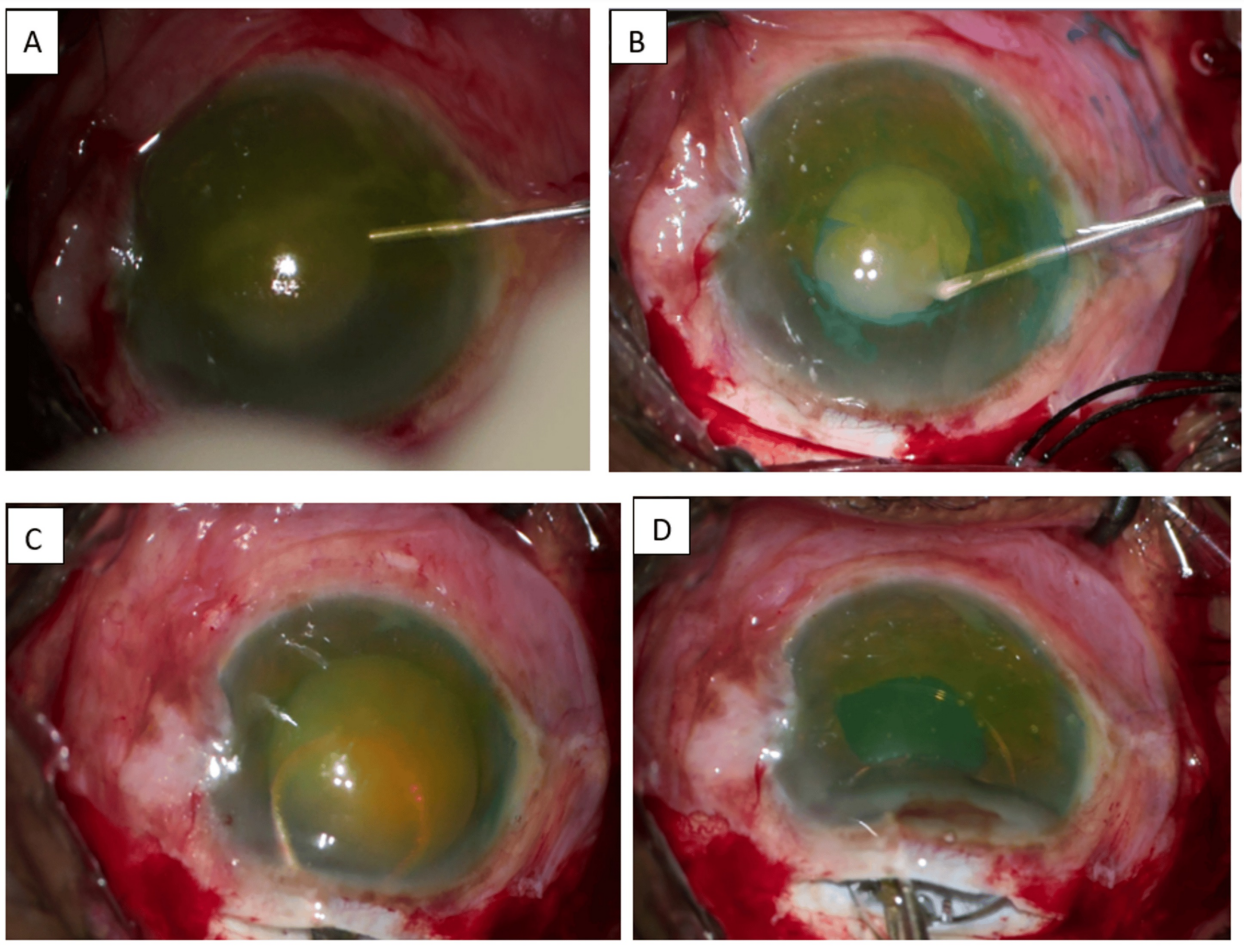
Intraoperative pictures taken through Zeiss Lumera 700 operating microscope (A) Washing leaked lens proteins with saline through the side port incision. (B) Milky white liquefied cortex oozing while making rhexis. (C) Nucleus delivery. (D) IOL implantation IOL: intraocular lens

Excessive capsular mobility was noted while performing capsulorrhexis, and the rhexis tear extended toward the periphery, suggestive of zonular compromise. The nucleus was carefully prolapsed into the anterior chamber and expelled through the sclerocorneal tunnel. Residual cortex was removed using aspiration with a Simcoe cannula. Because of compromised capsular support resulting from the broken anterior capsule and zonular dialysis, implantation of an intraocular lens within the capsular bag was not feasible. A three-piece intraocular lens was therefore implanted in the ciliary sulcus and centered. Intraoperative pictures depicting various surgical steps taken through the Zeiss Lumera 700 operating microscope (ZEISS Group, Oberkochen, Germany) are shown in Figure 2.

Intraoperative surgical manoeuvres are shown in Video 1.

**Video 1:** Intraoperative surgical steps (through Zeiss Lumera 700 surgical microscope)

**Figure 3:**
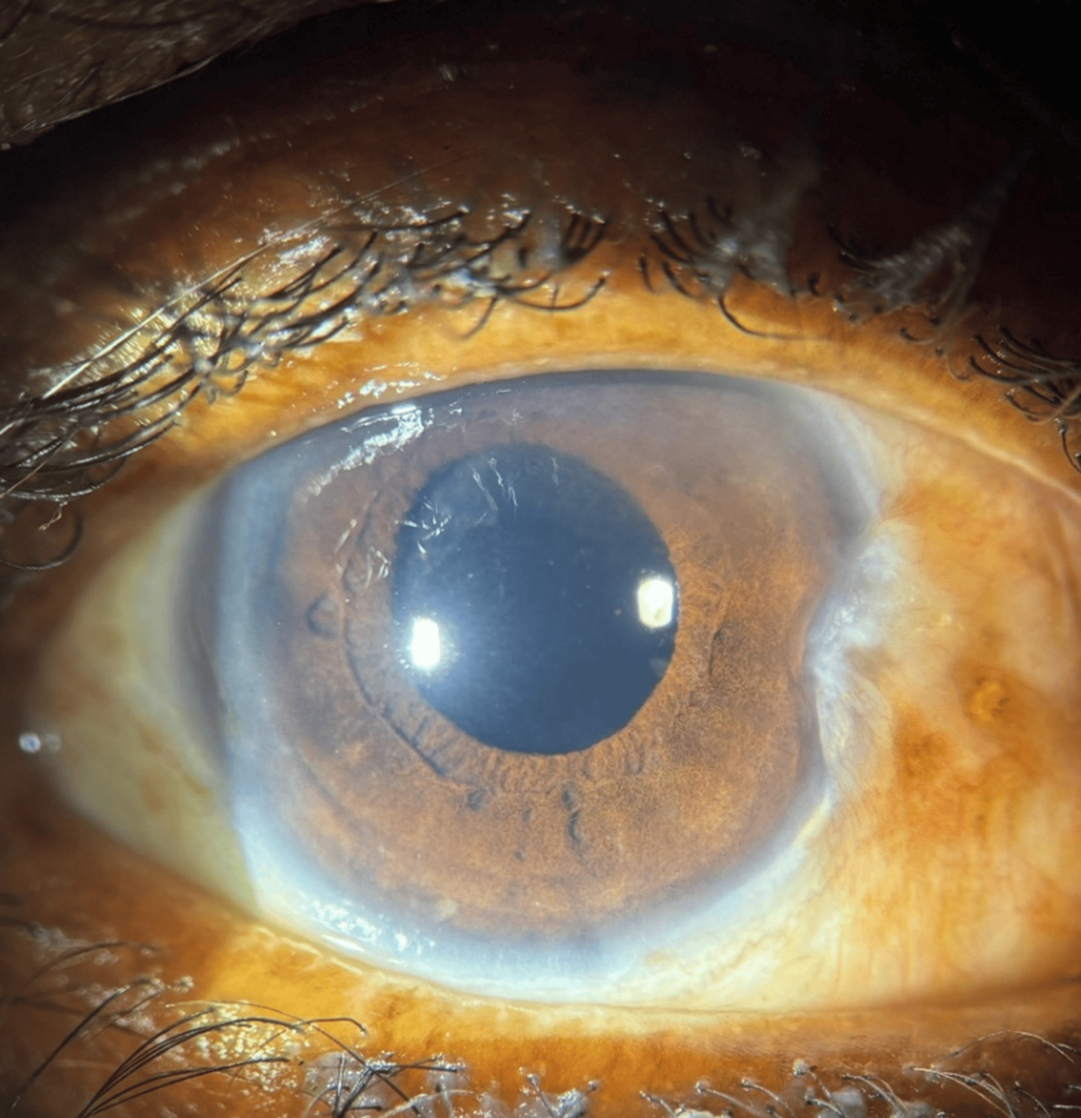
Postoperative image showing implanted intraocular lens

Postoperative treatment included topical moxifloxacin eye drops and prednisolone acetate eye drops. The postoperative image is shown in Figure 3.

The combination of timolol and brimonidine eye drops was continued to maintain intraocular pressure control. Postoperatively, the intraocular pressure decreased to 18 mmHg. Corneal clarity improved gradually, and the intraocular lens remained well-positioned. Visual acuity improved to 6/18 during the first postoperative week and further improved to 6/12 by the fourth postoperative week.

## Discussion

This report illustrates the classical presentation of phacolytic glaucoma secondary to hypermature cataract. Phacolytic glaucoma arises in hypermature cataract when increased lens capsule fragility allows leakage of high molecular weight lens proteins into the anterior chamber. These proteins incite macrophage phagocytosis; the resultant swollen macrophages obstruct the trabecular meshwork, impairing aqueous outflow and precipitating an acute rise in IOP [2]. Prompt recognition of this condition is important because sustained elevation of IOP can cause irreversible optic nerve damage. Initial treatment focuses on rapid reduction of IOP using hyperosmotic agents and topical aqueous suppressants. Once IOP has been controlled, definitive treatment involves removal of the cataractous lens, which eliminates the source of protein leakage and resolves the secondary glaucoma [3].

Surgical management of hypermature cataracts may be technically challenging due to capsular fragility, liquefied cortical material, and compromised zonular support. In such situations, capsule staining with trypan blue improves visualization and facilitates safer surgical manipulation. When capsular support is inadequate, as in this case, implantation of a three-piece intraocular lens in the ciliary sulcus provides a stable alternative to in-the-bag placement to avoid complications such as intraocular lens instability, decentration, tilt, and vitreous prolapse [5]. The novelty of this case lies in the presentation of a hypermature cataract complicated by phacolytic glaucoma, a condition that has become relatively uncommon in modern ophthalmic practice due to earlier cataract surgery. The coexistence of these conditions makes the case clinically significant. The patient presented with a markedly elevated IOP of 54 mmHg, representing a severe rise in pressure with a potential risk of optic nerve damage. Diagnostic evaluation was complicated by significant corneal edema and opacity, which obscured visualization of the anterior segment during initial examination.

The case was further complicated by intraoperative findings, including a small pupil, a broken anterior capsule, and zonular dialysis. These factors required modification of standard surgical techniques, including trypan blue staining, controlled prolapse of the nucleus into the anterior chamber, and sulcus placement of a three-piece intraocular lens. Despite these challenges, the patient achieved substantial visual recovery, highlighting the effectiveness of timely and tailored surgical management. This case has significant educational value because it demonstrates the classical clinical features of phacolytic glaucoma and highlights a hypermature cataract as an important cause of secondary glaucoma. Although lens-induced glaucomas are well described in ophthalmic literature, they are encountered less frequently today due to earlier cataract intervention. The case also reinforces the importance of prompt intraocular pressure control before undertaking cataract surgery. Rapid reduction of IOP is essential to prevent optic nerve damage and improve surgical conditions by reducing corneal edema.

From a surgical perspective, this report provides practical insight into the management of intraoperative complications such as capsular rupture and zonular dialysis in advanced cataracts. The use of adjunctive techniques such as capsule staining and alternative intraocular lens placement demonstrates strategies that may be useful in similar complex cases. Additionally, the case highlights the public health implications of delayed cataract treatment. In resource-limited settings, patients may present late with advanced disease and associated complications such as lens-induced glaucoma. Reporting such cases contributes valuable information to the global ophthalmology literature.

## Conclusions

Hypermature cataract with secondary glaucoma is a vision-threatening condition requiring prompt recognition and management. Early reduction of IOP followed by cataract extraction can lead to good visual outcomes. Even in the presence of advanced pathology and intraoperative challenges such as capsular rupture or zonular dialysis, careful surgical technique and appropriate intraocular lens placement can result in favorable recovery.
